# Promise and challenges of peptide-poly: ICLC vaccines for adult and pediatric gliomas

**DOI:** 10.1186/2051-1426-1-S1-P228

**Published:** 2013-11-07

**Authors:** Hideho Okada, Lisa H Butterfield, Ronald L Hamilton, Andres M Salazar, Douglas M Potter, Frank S Lieberman, Ian F Pollack

**Affiliations:** 1 University of Pittsburgh Cancer Institute, Pittsburgh, PA, USA; 2 Oncovir, Inc., Washington, DC, USA

## 

We currently run phase I studies of subcutaneous vaccinations with synthetic peptides for glioma-associated antigen (GAA) epitopes emulsified in Montanide-ISA-51 and intramuscular administration of poly-ICLC in HLA-A2+ adult and pediatric patients with gliomas. Primary endpoints were safety and CD8+ T-cell responses against vaccine-targeted GAAs: IL-13Rα2, EphA2, Survivin and WT1 (WT1 in adults only). Adults with WHO grade 2 low-grade glioma (LGG) have an extremely high risk for transformation to high-grade glioma (HGG), and most patients eventually die of the disease. Because patients with LGGs may not be as immuno-compromised as patients with HGG, they may exhibit greater immunological response to and benefit from the vaccines. We conducted a phase I vaccine study with: newly diagnosed high-risk LGG without prior radiation therapy (RT) (Cohort 1); newly diagnosed high-risk LGG with prior RT (Cohort 2); or recurrent LGG (Cohort 3). Cohorts 1, 2, and 3 have enrolled 12, 1, and 10 patients, respectively. No regimen-limiting toxicity has been encountered except for one case with Grade 3 fever (Cohort 1). Cohort 1 patients demonstrated significantly higher magnitude of IFN-γ ELISPOT responses than Cohort 3 patients for all 4 GAA epitopes, suggesting that newly diagnosed patients may have better vaccine-responsiveness than recurrent patients. The magnitude of the IFN-γ ELISPOT responses in this study is significantly higher than that observed in our previous phase I/II study in HGG patients. Median progression-free survival (PFS) periods are 21 months (Cohort 1; range 10-44) and 12 months (Cohort 3; range 3-28). In Cohort 1, 3 patients are still progression-free (32, 33 and 44 months to date). The only patient with large astrocytoma in Cohort 2 has been progression-free for over 54 months since diagnosis. There was a positive trend for IFN-γ ELISPOT responses and PFS. Diffuse brainstem gliomas (BSGs) and other HGGs of childhood carry a dismal prognosis. To date, 24 children were enrolled, 14 with newly diagnosed BSG treated with RT, and 10 with newly diagnosed BSG or HGG treated with RT and concurrent chemotherapy. No dose-limiting non-CNS toxicity was encountered. Five children had symptomatic pseudoprogression, which responded to corticosteroids and was associated with prolonged survival. Nineteen had stable disease for > 2 cycles, 2 had partial responses, and 1 had prolonged disease-free status after surgery. Median survival among the BSG cohort exceeded 13 months. ELISPOT analysis in 15 children showed GAA responses in 12, to IL-13Rα2 in 9, EphA2 in 7, and survivin in 7. Careful monitoring and management of pseudoprogression is warranted.

